# Temperature Changes in Poor-Grade Aneurysmal Subarachnoid Hemorrhage: Relation to Injury Pattern, Intracranial Pressure Dynamics, Cerebral Energy Metabolism, and Clinical Outcome

**DOI:** 10.1007/s12028-023-01699-0

**Published:** 2023-03-15

**Authors:** Teodor Svedung Wettervik, Anders Hånell, Elisabeth Ronne-Engström, Anders Lewén, Per Enblad

**Affiliations:** Section of Neurosurgery, Department of Medical Sciences, Uppsala University, 751 85 Uppsala, Sweden

**Keywords:** Aneurysmal subarachnoid hemorrhage, Cerebral microdialysis, Cerebral physiology, Neurointensive care, Temperature

## Abstract

**Background:**

The aim was to study the course of body temperature in the acute phase of poor-grade aneurysmal subarachnoid hemorrhage (aSAH) in relation to the primary brain injury, cerebral physiology, and clinical outcome.

**Methods:**

In this observational study, 166 patients with aSAH treated at the neurosurgery department at Uppsala University Hospital in Sweden between 2008 and2018 with temperature, intracranial pressure (ICP), and microdialysis (MD) monitoring were included. The first 10 days were divided into the early phase (days 1–3) and the vasospasm phase (days 4–10).

**Results:**

Normothermia (temperature = 36–38 °C) was most prevalent in the early phase. A lower mean temperature at this stage was univariately associated with a worse primary brain injury, with higher Fisher grade and higher MD glycerol concentration, as well as a worse neurological recovery at 1 year. There was otherwise no association between temperature and cerebral physiological variables in the early phase. There was a transition toward an increased burden of hyperthermia (temperature > 38 °C) in the vasospasm phase. This was associated with concurrent infections but not with neurological or radiological injury severity at admission. Elevated temperature was associated with higher MD pyruvate concentration, lower rate of an MD pattern indicative of ischemia, and higher rate of poor neurological recovery at 1 year. There was otherwise no association between temperature and cerebral physiological variables in the vasospasm phase. The associations between temperature and clinical outcome did not hold true in multiple logistic regression analyses.

**Conclusions:**

Spontaneously low temperature in the early phase reflected a worse primary brain injury and indicated a worse outcome prognosis. Hyperthermia was common in the vasospasm phase and was more related to infections than primary injury severity but also with a more favorable energy metabolic pattern with better substrate supply, possibly related to hyperemia.

**Supplementary Information:**

The online version contains supplementary material available at 10.1007/s12028-023-01699-0.

## Introduction

Aneurysmal subarachnoid hemorrhage (aSAH) is a severe disease with high mortality and poor neurological recovery [1]. Basic management includes prevention of rebleeding by early aneurysm occlusion, cerebrospinal fluid diversion in case of acute hydrocephalus, and prevention and treatment of delayed ischemic neurological deficits (DINDs) [2].

In addition, neurointensive care (NIC) is important to optimize cerebral physiology to avoid secondary brain injury [3–6]. The role of body temperature has received great interest in NIC management of aSAH, although its net effects on the brain and optimal treatment have remained inconclusive [2, 7]. First, it seems that patients with aSAH exhibit a biphasic temperature pattern. Particularly for poor-grade aSAH, temperature initially drops the first hours from ictus, and thereafter hyperthermia gradually ensues [8]. This disturbance in temperature has been partly ascribed to a greater burden of subarachnoid hemorrhage (SAH) and IVH [9], possibly because of a change in temperature set point from toxic and inflammatory effects and/or ischemic complications in the brainstem/hypothalamus [10]. Hyperthermia is also related to infectious complications that follows during NIC. Several studies indicate that hyperthermia induces negative cerebral effects, including increased cerebral energy metabolism, cerebral hyperemia, blood–brain barrier disruption, cerebral edema, intracranial hypertension, and excitotoxicity [11–13]. Correspondingly, hyperthermia has been associated with the development of DIND [9, 14] and worse neurological recovery [3, 9, 14, 15]. However, it remains elusive if these associations are causal or mere epiphenomena of a worse primary and secondary brain injury.

Although several studies on body temperature in aSAH have been conducted, there is still a paucity of publications on high-frequency multimodality monitoring data in this matter. Consequently, in this study, we aimed to investigate the course of body temperature in the acute phase of poor-grade aSAH in relation to the primary brain injury severity, cerebral physiology, clinical course, and long-term outcome.

## Methods

### Patients

Patients with aSAH admitted to the Department of Neurosurgery, Uppsala University Hospital, Sweden, between 2008 and 2018 were eligible for this study. Of 605 patients with SAH, 194 were monitored with body temperature, intracranial pressure (ICP), and cerebral microdialysis (MD). Of these 194 patients, 22 who had an etiology (arteriovenous malformation or angiogram-negative SAH) other than aneurysmal hemorrhage and six patients who developed early brain death were excluded. Consequently, the study cohort was based on 166 patients with aSAH.\

### Management

Our aSAH management protocol has been described in detail in previous studies [3, 4, 16, 17]. In short, treatment goals were ICP ≤ 20 mm Hg, cerebral perfusion pressure (CPP) ≥ 60 mm Hg, systolic blood pressure ≥ 100 mm Hg, pO_2_ > 12 kPa, arterial glucose concentration 5–10 mM, electrolytes within normal ranges, and temperature < 38 °C.

Intracranial aneurysms were treated with early occlusion by endovascular embolization or open surgical interventions. All patients received nimodipine after admission. Intubation and mechanical ventilation were indicated in unconscious patients not obeying commands. Propofol and morphine were given for sedation and analgesia, respectively. An external ventricular drain (EVD) was used for ICP monitoring in intubated patients. The EVD was opened at a drainage level of 15 mm Hg, if ICP remained above 20 mm Hg. Thiopental infusion and decompressive craniectomy were only used in cases of refractory elevated ICP.

DIND was defined as a new onset of focal neurological deficit or deterioration in consciousness not explained by, for example, hydrocephalus, rebleeding, or meningitis. If a manifest cerebral infarction could not be detected on computed tomography, a modest hypertension, hypervolemia, and hemodilution (HHH) therapy was initiated. HHH therapy included supine position, colloid fluids to increase the intravascular volume using albumin 20% and dextran solutions with near-zero fluid balance, and moderately elevated systolic blood pressure target above 140 mm Hg and depending on blood pressure at the start of HHH therapy [18, 19]. If this was not sufficient, the blood pressure was increased further, followed by intraarterial nimodipine and balloon angioplasty if necessary.

Fever (temperature > 38 °C) was treated with 1 g of paracetamol four times per day and surface cooling using cooling blankets and body-shaped wrap cover dressing devices. Induced hypothermia was not a part of our treatment protocol.

### Data Acquisition

All physiological data were collected at 100 Hz using the Odin software [20]. Body temperature was monitored with a probe in the urinary catheter (Mon-a-ThermTM Foley catheter with temperature sensor 400TM, Covidien, Germany) [13]. ICP was monitored with an EVD system (HanniSet, Xtrans, Smith Medical GmbH, Glasbrunn, Germany). ICP was monitored using the Xtrans pressure transducer (CODAN pvb Medical GmbH, Germany). When the EVD was opened, the ICP monitoring continued and the drainage level was adjusted to achieve the prescribed ICP, usually 15 mm Hg. The EVD has a nonreturn duck-bill valve, and the outflow resistance of the valve preserves the pulse wave to some extent when the system is opened, although the amplitudes and morphologies change [21]. ICP slow waves seem to be preserved when the EVD is opened [21, 22], which supports that the pressure reactivity index (PRx) is still reliable. Arterial blood pressure was monitored invasively in the radial artery at heart level. PRx was calculated as the 5-min correlation of 10-s averages of ICP and mean arterial blood pressure [23, 24]. Mean daily values of ICP, mean arterial blood pressure, CPP, and PRx were calculated for the first 10 days after ictus.

Cerebral energy metabolism was monitored with the 70 High Cut-Off MD catheter with a membrane length of 10 mm and a membrane cutoff of 20 kDa (M Dialysis AB, Stockholm, Sweden). The MD catheter was placed via a burr hole, adjacent to the EVD in normal-appearing brain tissue in the frontal lobe. As previously described [17], the MD was perfused by means of a microinjection pump (106 MD Pump, M Dialysis AB) at a rate of 0.3 µL/min with sterile artificial cerebrospinal fluid containing NaCl 147 mM, KCl 2.7 mM, CaCl_2_ 1.2 mM, and MgCl_2_ 0.85 mM. Cerebral interstitial glucose, pyruvate, lactate, glycerol, glutamate, and urea concentrations were estimated hourly using a CMA 600 analyzer or the ISCUSflex Microdialysis Analyzer (M Dialysis AB). MD glycerol and MD glutamate estimates were missing in 106 patients when the CMA 600 was used. MD urea was monitored to validate catheter performance [25]. Total imprecision coefficient of variation was < 10% for all analytes.

The percentages of good monitoring time with episodes of hypothermia (temperature < 36 °C), normothermia (temperature = 36–38 °C), hyperthermia (temperature > 38 °C), ICP elevation (above 20 mm Hg), and low CPP (below 60 mm Hg) were calculated in the early phase (days 1 to 3) and the vasospasm phase (days 4 to 10). The thresholds were chosen in accordance with our management protocol.

Furthermore, the energy metabolic pattern was classified as “ischemia” (MD LPR > 25 and concurrent MD pyruvate < 120 µM) or “cerebral mitochondrial dysfunction” (MD LPR > 25 and concurrent MD pyruvate > 120 µM). The MD LPR threshold of 25 for metabolic disturbances was chosen in accordance with the consensus statement from 2014 [26]. The MD pyruvate threshold of 120 µM was chosen because this is the highest pyruvate value for ischemic cerebral conditions and the lowest value for nonischemic cerebral conditions according to previous studies [27, 28]. The percentages of monitoring time in these energy metabolic states were calculated for the early phase and the vasospasm phase based on hourly MD data.

Mean values and/or the percentages of monitoring time above/below the thresholds mentioned above were calculated for each phase in the Odin software.

### Outcome

Clinical outcome was assessed 12 months after ictus using the Extended Glasgow Outcome Scale (GOS-E) [29, 30] by trained personnel using structured telephone interviews. The GOS-E scale has eight categories of outcome and ranges from death (1) to upper good recovery (8). Clinical outcome was dichotomized as favorable/unfavorable (GOS-E 5–8/1–4).

### Statistical Analysis

The analysis aimed to determine primarily the association of temperature with cerebral physiology and secondarily the association of these insults with DIND and clinical outcome.

Nominal variables were described as numbers or proportions, and ordinal and continuous variables were described as medians with interquartile range (IQR). The associations of temperature with ICP, CPP, PRx, MD variables, DIND, and clinical outcome were evaluated with univariate analysis (Spearman) for the early phase and the vasospasm phase. For those cerebral physiological variables that were univariately associated with temperature for the specific phases (early or late), a multiple linear regression analysis was performed using age, World Federation of Neurosurgical Societies (WFNS) grade, and temperature as independent variables and the specific cerebral physiological variable as the dependent variable. In these regressions, the dependent variable MD ischemia was log_10_-transformed because of skewness in its distribution. A multiple logistic regression analysis for favorable outcome was conducted with age, WFNS grade, and temperature as independent variables. Missing data were excluded from the analyses. A *p* value < 0.05 was considered statistically significant. Because this was an exploratory study, we abstained from multiple corrections. The statistical analyses were performed in SPSS version 28 (IBM Corp, Armonk, NY).

### Ethics

All procedures performed in the studies involving humans were in accordance with the ethical standards of the institutional and national research committee and with the 1964 Helsinki Declaration and its later amendments. The study was approved by the Swedish Ethical Review Authority (Dnr 2020–05462).

## Results

### Demography, Admission Variables, Treatments, and Clinical Outcome

Of 166 patients with aSAH, 111 (67%) were female, and the median age was 59 (IQR 51–67) years (Table 1). At admission, the median WFNS grade was 4 (IQR 2–4) and the median Fisher grade was 4 (IQR 3–4). The intracranial aneurysm was located in the anterior rather than the posterior circulation in most cases (79% vs. 21%). The aneurysms were mostly occluded with endovascular embolization (78%), less often occluded with clipping (19%), and occasionally occluded with both techniques (1%), and in a few cases, no treatment was possible (2%). In total, 43 (26%) developed DIND, 18 (11%) received thiopental, and 19 (11%) were treated with decompressive craniectomy. In addition, 99 (60%) patients developed an infection (culture-positive) and 143 (86%) patients were treated with antibiotics during the first 10 days after ictus. Nineteen (11%) exhibited an infection both in the early phase and in the vasospasm phase, and 99 (60%) exhibited an infection only in the vasospasm phase. Eight (5%) developed culture-positive EVD-related meningitis, and 93 (56%) developed a culture-positive systemic (respiratory or urinary tract) infection. At 1 year post ictus, the median GOS-E score was 3 (IQR 3–5).

**Table 1:** Demography, admission variables, treatments, and clinical outcome

	Value
Patients, *n* (%)	166 (100%)
Age, median (IQR)	59 (51–67)
Sex (male/female), *n* (%)	55/111 (33%/67%)
WFNS grade, median (IQR)	4 (2–4)
Fisher grade, median (IQR)	4 (3–4)
Anterior/posterior aneurysm location, *n* (%)	131/35 (79%/21%)
No aneurysm treatment/embolization/clipping/both, *n* (%)	4/130/31/1 (2%/78%/19%/1%)
DIND, *n* (%)	43 (26%)
Thiopental, *n* (%)	18 (11%)
Decompressive craniectomy, *n* (%)	19 (11%)
GOS-E, median (IQR)	3 (3–5)

DIND, delayed ischemic neurological deficits, GOS-E, Glasgow Outcome Scale-Extended, IQR, interquartile range, WFNS, World Federation of Neurosurgical Societies

### Temperature and Cerebral Physiology

**Fig. 1:**
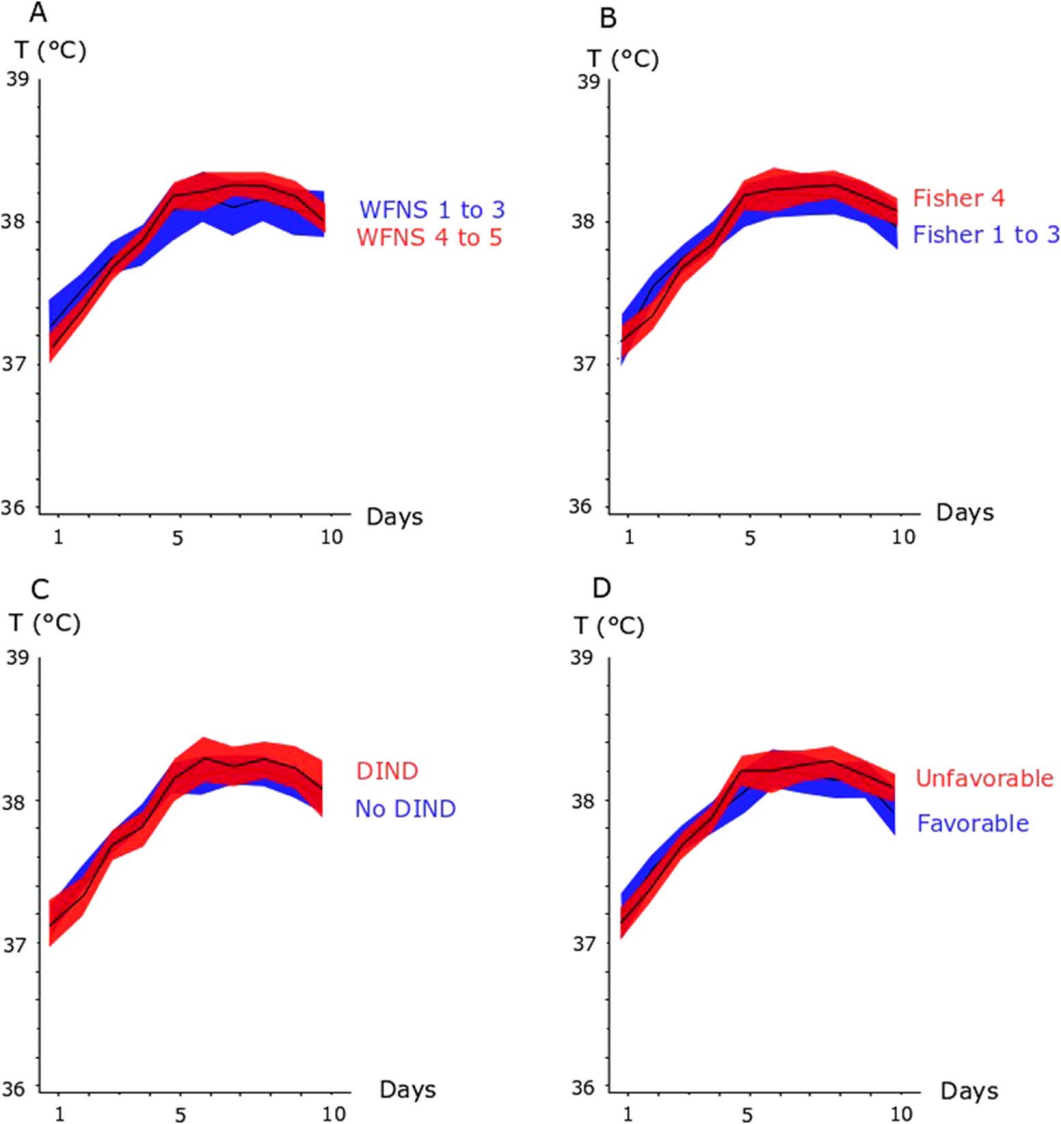
Temporal course of body temperature in the acute phase after aSAH in relation to neurological injury and radiological injury severity, development of DIND, and clinical outcome. The figure demonstrates the temporal course of mean daily body temperature (95% CI) in relation to neurological (**a**) and radiological (**b**) injury severity at admission, the risk of developing DIND (**c**), and long-term clinical outcome (**d**). aSAH aneurysmal subarachnoid hemorrhage, CI confidence interval, DIND delayed ischemic neurological deficits, WFNS World Federation of Neurosurgical Societies

Temperature and the cerebral physiological variables are described in detail in Table 2, Fig. 1, and Supplementary Fig. 1. Temperature was significantly higher in the vasospasm phase compared with the early phase. From the early phase to the vasospasm phase, the burden of ICP > 20 mm Hg decreased, the burden of CPP < 60 mm Hg decreased, and pressure autoregulation worsened (higher PRx). For the MD variables, MD pyruvate and MD lactate concentrations increased, MD ischemia decreased, and MD mitochondrial failure increased. MD glucose, MD glycerol, and MD glutamate concentrations remained unchanged.

**Table 2:** Temperature and cerebral physiology in the early phase and the vasospasm phase

	Early phase	Vasospasm phase
Body temperature (°C)	37.5 (37.3–37.8)***	38.2 (37.9–38.4)***
Hypothermia (%)	5 (0–12)***	0 (0–1)***
Hyperthermia (%)	5 (0–21)***	57 (34–77)***
ICP (mm Hg)	12 (10–14)	11 (9–14)
ICP > 20 mm Hg (%)	2 (1–5)***	2 (1–3)***
MAP (mm Hg)	87 (83–92)***	93 (88–100)***
CPP (mm Hg)	75 (71–81)***	82 (77–89)***
CPP < 60 mm Hg (%)	3 (1–8)***	1 (0–3)***
PRx (coefficient)	0.15 (0.07–0.24)**	0.19 (0.10–0.29)**
MD glucose (mM)	2.2 (1.5–3.0)	2.1 (1.4–3.0)
MD pyruvate (µM)	124 (93–163)***	161 (124–200)***
MD lactate (mM)	3.3 (2.3–5.6)***	4.3 (3.1–6.0)***
MD LPR (ratio)	26 (20–40)	26 (21–33)
MD ischemia (%)	13 (0–50)***	1 (0–11)***
MD mitochondrial failure (%)	11 (0–47)***	26 (2–69)***
MD glycerol (µM)	103 (60–202)	88 (61–139)
MD glutamate (µM)	16 (6–69)	18 (2–76)

MD glycerol and MD glutamate estimates were missing in 106 patients when the MD samples were analyzed with the CMA 600 instead of ISCUSflex. Hypothermia = temperature < 36 °C. Hyperthermia = temperature > 38 °C. MD mitochondrial failure = MD LPR > 25 and MD pyruvate > 120 µM. MD poor substrate supply = MD LPR > 25 and MD pyruvate < 120 µM. All values are medians (IQR). Asterisks indicate statistical significance

CPP, cerebral perfusion pressure, ICP, intracranial pressure, IQR, interquartile range, LPR, lactate-to-pyruvate ratio, MAP, mean arterial blood pressure, MD, microdialysis, PRx, pressure reactivity index

*p < 0.05; **p < 0.01; ***p < 0.001

### Temperature in Relation to Injury Severity, Infections, Intracranial Pressure Dynamics, and Cerebral Energy Metabolism

Patients with a higher Fisher grade exhibited lower temperature in the early phase (*r* =  − 0.16, *p* < 0.05), but no association with temperature was found in the vasospasm phase (Table 3). No association between WFNS grade and temperature was found in any phase. Patients with concurrent infections in the vasospasm period exhibited slightly higher temperature in that phase (*r* = 0.20, *p* < 0.05) (Table 3).

**Table 3:** Systemic body temperature in relation to injury characteristics, clinical course, cerebral physiology, and clinical outcome: a Spearman correlation analysis

Variables	Early phase	Vasospasm phase
Infection (yes)	0.03	0.20*
WFNS (grade)	− 0.13	0.07
Fisher (grade)	− 0.16*	0.02
DIND (yes)	− 0.07	0.04
ICP > 20 mm Hg (%)	0.09	− 0.09
CPP < 60 mm Hg (%)	0.01	0.03
PRx (coefficient)	− 0.02	0.04
MD glucose (mM)	− 0.03	0.09
MD pyruvate (µM)	− 0.04	0.21*
MD lactate (mM)	− 0.04	0.05
MD LPR (coefficient)	− 0.06	− 0.16
MD ischemia (%)	− 0.06	− 0.26**
MD mitochondrial failure (%)	− 0.02	− 0.11
MD glycerol (µM)	− 0.35**	− 0.09
MD glutamate (µM)	0.04	0.04
GOS-E (scale)	0.22**	− 0.16*

MD glycerol and MD glutamate estimates were missing in 106 patients when the MD samples were analyzed with the CMA 600 instead of ISCUSflex. MD mitochondrial failure = MD LPR > 25 and MD pyruvate > 120 µM. MD poor substrate supply = MD LPR > 25 and MD pyruvate < 120 µM. Asterisks indicate statistical significance

CPP, cerebral perfusion pressure, DIND, delayed ischemic neurological deficits, GOS-E, Glasgow Outcome Scale-Extended, ICP, intracranial pressure, LPR, lactate-to-pyruvate ratio, MD, microdialysis, PRx, pressure reactivity index, WFNS, World Federation of Neurosurgical Societies

^*^p < 0.05; **p < 0.01; ***p < 0.001

ICP and CPP insults were not associated with temperature. A higher MD glycerol concentration (*r* =  − 0.35, *p* < 0.01) was associated with lower temperature in the early phase (Table 3). In addition, higher MD pyruvate concentration (*r* = 0.21, *p* < 0.05) and a lower rate of MD ischemia (*r* =  − 0.26, *p* < 0.01) correlated with higher temperature in the vasospasm period (Table 3). If the thiopental cases were excluded, higher body temperature was also associated with higher MD pyruvate concentration (*r* = 0.21, *p* < 0.05) and lower LPR (*r* =  − 0.19, *p* < 0.05), but there were otherwise no difference in these correlation analyses.

In a multiple linear regression analysis, higher temperature (*β* =  − 0.23, *p* < 0.05) in the vasospasm phase independently predicted lower rate of MD ischemia after adjustment for age and WFNS grade (Table 4), although the regression was only marginally significant (*p* = 0.058). In a similar regression with MD pyruvate as the dependent variable, higher temperature (*β* = 0.18, *p* < 0.05) was associated with higher MD pyruvate concentration, but the regression was not significant (*p* = 0.17). Lastly, a similar regression was conducted with MD glycerol as the dependent variable in the early phase, but neither temperature nor the regression as a whole was significant.

**Table 4:** Association between MD ischemia and temperature: a multiple linear regression analysis

	MD pyruvate		MD ischemia	
Variables	*β*	*p* value	*β*	*p* value
Age	0.03	0.70	− 0.14	0.22
WFNS	− 0.03	0.69	0.19	0.10
Temperature	0.18	**0.03**	** − 0.23**	**0.04**

The variable MD ischemia was log_10_-transformed because of skewness in its distribution. Regression 1, MD pyruvate: *R*^2^ = 0.04, ANOVA *p* = 0.17. Regression 2, MD ischemia: *R*^2^ = 0.10, ANOVA *p* = 0.058

Bold indicates statistical significance

ANOVA, analysis of variance, MD, microdialysis, WFNS, World Federation of Neurosurgical Societies

### Temperature in Relation to DIND and Clinical Outcome

Development of DIND was not associated with body temperature in any phase (*p* > 0.05). A lower GOS-E score was associated with lower temperature in the early phase and higher temperature in the vasospasm phase (Table 3). The associations between temperature and clinical outcome did not hold true after adjustment for age and WFNS grade in a multiple logistic outcome regression (Table 5). Higher age and WFNS grade were independent predictors of a lower rate of favorable outcome in the regressions.

**Table 5:** Body temperature in the early phase and the vasospasm phase in relation to favorable outcome: multiple logistic outcome regression analyses

Variables	Early phase		Vasospasm phase	
	OR (95% CI)	*p* value	OR (95% CI)	*p* value
Age (years)	0.97 (0.93–1.00)	0.06	0.96 (0.93–0.99)	**0.01**
WFNS (grade)	0.57 (0.43–0.77)	**0.001**	0.57 (0.43–0.76)	**0.001**
Body temperature (°C)	1.07 (0.34–3.41)	0.91	0.73 (0.30–1.77)	0.49

Bold indicates statistical significance

CI, confidence interval, OR, odds ratio, WFNS, World Federation of Neurosurgical Societies

## Discussion

In this study of 166 patients with aSAH with temperature, ICP, and MD monitoring, we found that those with higher Fisher grade, elevated cerebral MD injury biomarkers (glycerol), and worse long-term outcome spontaneously exhibited lower mean body temperature early after ictus. This could be an endogenous neuroprotective mechanism or a hypothalamic disturbance in temperature set point following a more severe aSAH. In the vasospasm phase, on the other hand, most patients developed hyperthermia. Higher temperature was associated with infectious complications and a cerebral state of better energy substrate supply but not with worse primary brain injury (WFNS/Fisher grade), ICP/CPP insults, or development of DIND. The association with less ischemia/better cerebral substrate supply according to the MD indicates a hyperthermia-induced hyperemia predisposing for increased substrate delivery. Still, higher body temperature in the vasospasm phase correlated with worse outcome, but this did not hold true in multiple regressions.

### Temperature Changes After aSAH: Relation to Injury Severity and Infectious Complications

In this study, most patients exhibited normothermia and to some extent spontaneous hypothermia in the early phase. Those with a lower mean body temperature had a more severe primary brain injury, as indicated by radiological (Fisher) and cerebral MD (higher glycerol concentration) variables. This is interesting and has previously to our knowledge only been documented by Takagi et al. [8], who found that early spontaneous hypothermia is in fact frequent in poor-grade aSAH cases and is probably a consequence of more severe brain damage that also involves hypothalamic and brainstem areas responsible for temperature control. In addition, sedative agents typically lower temperature and are usually administered to these patients with poor-grade aSAH. These agents might have also influenced the results.

Subsequently, after 3–4 days post ictus, there was a shift toward higher temperature to hyperthermic levels (above 38 °C) for most patients in our study. The gradual development of hyperthermia is well known and has classically been attributed to infectious complications during NIC and neurogenic mechanisms post aSAH. We found a weak but significant association between temperature and presence of concurrent infections, in line with previous notions. Neurogenic hyperthermia typically occurs in poor-grade aSAH cases, but surprisingly, temperature was not associated with higher WFNS nor Fisher grade in the vasospasm phase in our cohort. One plausible reason for the lack of association could be the inclusion criteria, which led to a selection of patients who all exhibited a severe injury necessitating ICP and MD monitoring. Still, in other studies with different inclusion criteria and patient cohorts, neurogenic hyperthermia has been associated with poor-grade aSAH (higher Hunt and Hess/WFNS grade) and greater burden of SAH and IVH [9, 31]. In conclusion, patients with poor-grade aSAH seem to exhibit a biphasic temperature pattern with temperature in the lower range in the early phase, which is followed by hyperthermia in the vasospasm phase.

### Temperature in Relation to Cerebral Physiology in aSAH

There was no association between temperature and ICP in this study. This is in contrast to several previous studies [12, 32, 33], and the supposed mechanism is that hyperthermia increases energy metabolism, which in turn increases cerebral blood flow (CBF), cerebral blood volume (CBV), and consequently ICP. However, slight increases in CBV only yield significant ICP increases when ICP is already high and intracranial compliance is low. Because ICP was actively treated and the general use of open EVD led to a relatively high intracranial compliance, this could explain the lack of impact of any hyperthermia-induced increase in CBV on ICP in our study. We also used a slightly different analytical approach by assessing physiological variables averaged over longer episodes of time in this study rather than looking at brief episodes of fever. This might have reduced the chances to detect short-term effects on ICP before active ICP-lowering treatments had been initiated. Correspondingly, we also found no association between temperature and CPP.

Because body temperature is expected to induce CBF elevations to meet metabolic demand (increased CMRO_2_) [18, 32, 33], this could exhaust the distal cerebral vasodilatory reserve for patients with aSAH with concurrent proximal vasospasm and thereby disturb pressure cerebral autoregulation and CBF [34]. However, no association was found between temperature and PRx in this study. Instead, our findings indicate that higher temperature in the vasospasm phase was associated with a state of better cerebral substrate supply/less ischemia according to the MD. This is interesting considering that the net effect on cerebral energy metabolic delivery/demand from hyperthermia is not obvious. We have previously found that higher body temperature is associated with higher CBF and cerebral oxygen delivery in aSAH [18]. Stocchetti et al. found in a mixed brain injury cohort (spontaneous and traumatic intracranial bleedings) that episodes of elevated brain temperature correlated with a lower cerebral arteriovenous oxygen difference, higher brain tissue oxygenation, and otherwise unchanged cerebral substrate supply, ultimately most consistent with hyperemia with uncoupled energy supply/demand [33]. However, Oddo et al. found that hyperthermia was associated with higher LPR in a cohort of 18 patients with aSAH [12]. In addition, certain aSAH patient subgroups may exhibit different energy metabolic patterns of, for example, hyperglycolysis and mitochondrial dysfunction [35–37], and it could be speculated if hyperthermic patients exhibited a more efficient energy metabolism. Altogether, it seems that hyperthermia could both deplete the cerebral energy reserve by increased turnover and contribute to hyperemia and better substrate supply. Multimodality monitoring could therefore be useful to determine the cerebral net effects of changes in body temperature.

### Temperature in Relation to DIND and Clinical Outcome in aSAH

There was no association between body temperature and DIND in this study. Higher mean body temperature in the early phase did correlate with better outcome, whereas higher mean temperature in the vasospasm phase correlated with worse outcome, although none of these associations held true in multiple regression analyses. Previous studies on body temperature in relation to DIND and outcome are conflicting. Hyperthermia has been associated with radiographic vasospasm, as evaluated with digital subtraction angiography and transcranial Doppler [14, 38], but not in all studies [15]. Hyperthermia has also been independently associated with DIND in some studies [14, 31, 39] but not consistently [11, 38, 40]. Similarly, higher body temperature has been independently associated with worse outcome in many studies [9, 14, 31] but not all [3, 38]. Several factors could explain these discrepancies in the literature. Particularly, differences in patient cohorts (size and proportion of good-grade vs. poor-grade), definition of hyperthermia and method of temperature monitoring (ear, axillary, urinary catheter, brain, etc.), temperature management, and statistical analyses (different temporal phases post ictus and mean values vs. number of insults) contribute to study heterogeneity. In addition, although cerebral vasospasm, DIND, unfavorable outcome, and hyperthermia usually co-occur in poor-grade aSAH, it remains challenging to determine causality or whether they are epiphenomena of disease progression.

Hence, the potential dangers of hyperthermia are not fully confirmed in aSAH and there is consequently a lack of firm guideline recommendations for temperature management. The Neurocritical Care Society suggests avoiding hyperthermia (without any strict threshold), particularly in the vasospasm phase, by using paracetamol and nonsteroidal anti-inflammatory drugs at first hand and surface cooling and intravascular devices at second hand, while avoiding shivering [2]. The American Heart Association is vaguer and suggests avoiding hyperthermia in general by using standard treatments, without any further specifications [7]. Specifically, among the pharmacological agents, nonsteroidal anti-inflammatory drugs may reduce fever (but could simultaneously induce cerebral vasoconstriction) and worsen CPP and brain tissue oxygenation, predisposing for negative net effects on the brain in addition to risks of systemic complications [41]. Among the various cooling techniques, intravascular cooling shows some promise at least in traumatic brain injury, as initiation of cooling improves cerebral energy metabolism [42]. On balance, there is still a lack of high-quality evidence on the role of hyperthermia and optimal temperature management in aSAH.

### Methodological Considerations

There were several strengths with this study. First, it was relatively large and included 166 patients with high-resolution multimodality monitoring data. Second, we took into account temporal differences by assessing the early phase and vasospasm phase separately.

There were also some limitations. First, the inclusion criterion of multimodality monitoring led to some selection bias toward the most severe aSAH cases, which limits the external validity of this study. Second, body and brain temperature are highly correlated [43], but temperature may be as much as 1–2 °C higher in the brain [44]. This difference may reflect increased cerebral heat production as well as poor heat exchange from the brain. Interestingly, body temperature is not only an underestimation of brain temperature, but recent evidence also suggests that a greater difference between the two indicates increased energy production, better mitochondrial function, and greater chances of favorable outcome [45]. The role of brain temperature in aSAH clearly deserves further studies. Third, MD glycerol and MD glutamate estimates were missing in 106 patients when the MD samples were analyzed with the CMA 600 instead of ISCUSflex. This limits the reliability of the analyses of these variables. Fourth, hyperthermia was actively treated, and the concurrent treatment intensity of paracetamol, active cooling, and sedation likely influenced the results. For example, active cooling may lead to a detrimental stress response in some patients that may worsen the energy metabolic state. Fourth, the correlations were overall weak between, for example, body temperature and cerebral energy metabolism. This was expected, considering the plethora of systemic and cerebral variables that interact and influence each other. Fifth, we did not have access to granular data of hyperthermia treatments. In future efforts, it would be interesting to study in greater depth the effects of pharmacological and cooling treatments on cerebral physiology.

## Conclusions

The associations between temperature and injury severity, cerebral physiology, DIND, and clinical outcome are complex. In patients with poor-grade aSAH, lower body temperature in the early course may reflect a more severe primary brain injury and indicate a worse prognosis. Hyperthermia gradually ensues in the vasospasm phase and is associated with infectious complications. Although hyperthermia could deplete the cerebral energy metabolic reserve, higher temperature in this study correlated with an MD pattern indicative of better cerebral substrate supply/less ischemia. Multimodality monitoring may be useful to determine the cerebral net effects of changes in body temperature.

## Supplementary Information

Below is the link to the electronic supplementary material.

Supplementary file1 (DOCX 54 KB)
