# Supplementary material for: Temperature Changes in Poor-Grade Aneurysmal Subarachnoid Hemorrhage: Relation to Injury Pattern, Intracranial Pressure Dynamics, Cerebral Energy Metabolism, and Clinical Outcome
Source: Neurocrit Care. 2023 Mar 15;39(1):145–54. doi: 10.1007/s12028-023-01699-0 (PMC10499919; doi:10.1007/s12028-023-01699-0)
Supplement: Supplementary file 1 — Supplementary file1 (DOCX 54 KB) [file 12028_2023_1699_MOESM1_ESM.docx]

**Supplementary figure 1A-D. ICP, MAP, CPP and PRx the first 10 days post-ictus after aSAH
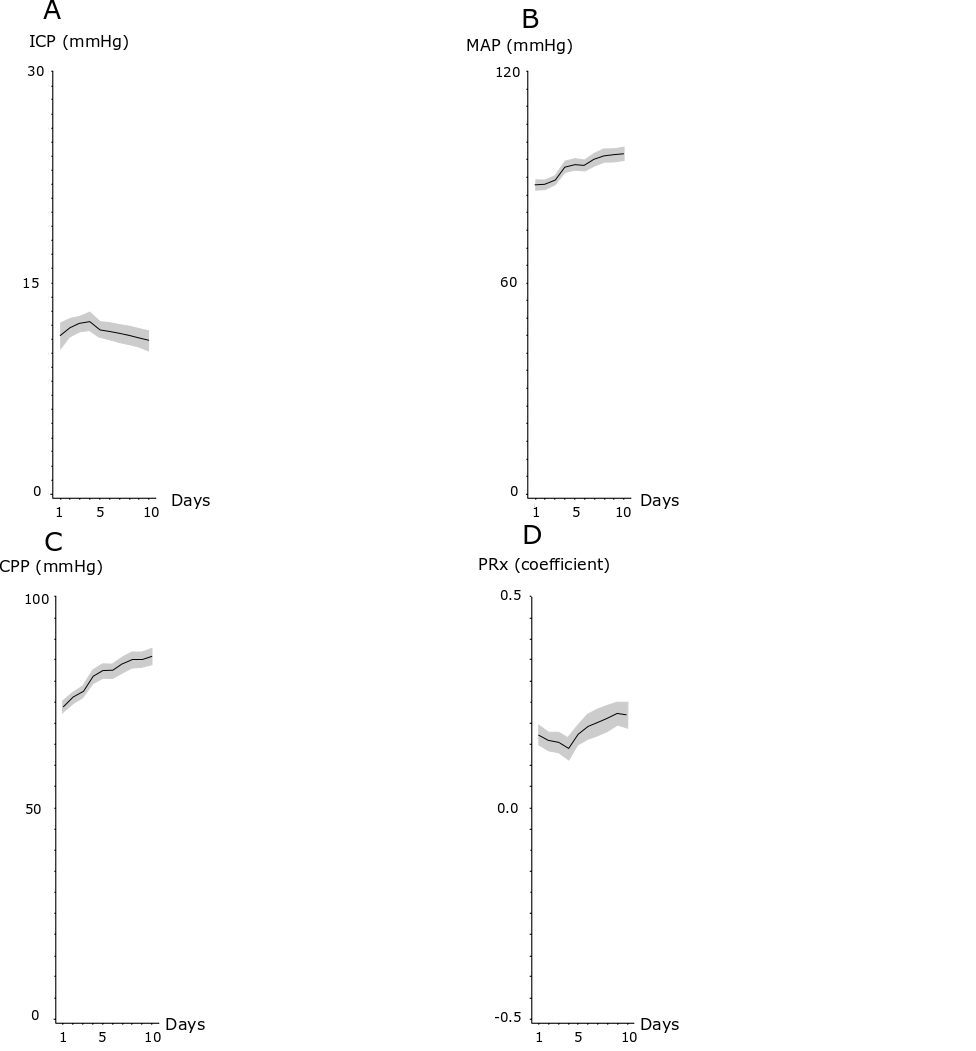
**

The figure demonstrates the temporal course of mean (95% CI) daily values for ICP (1A), MAP (1B), CPP (1C), and PRx (1D) the first 10 days after aSAH.

aSAH = Aneurysmal subarachnoid hemorrhage. CI = Confidence interval. CPP = Cerebral perfusion pressure. ICP = Intracranial pressure. MAP = Mean arterial blood pressure. PRx = Pressure reactivity index. WFNS = World federation of neurosurgical societies
